# In memory of Arild Vaktskjold MPH, DrScient (1962-2019)

**DOI:** 10.1080/22423982.2019.1656862

**Published:** 2019-08-27

**Authors:** Jon Oyvind Odland, Evert Nieboer

**Affiliations:** a Global Health, Norwegian University of Science and Technology, Trondheim, Norway; b International Health, Faculty of Health Sciences and Department of Community Medicine, The Arctic University of Norway, Tromsø, Norway; c Emeritus, McMaster University, Hamilton, Canada; d Toxicology, McMaster University, Hamilton, Canada; e Research & Graduate Student Advisor, Department of Community Medicine, UiT The Arctic University of Norway, Tromsø, Norway

**Keywords:** In memory of Arild Vaktskjold


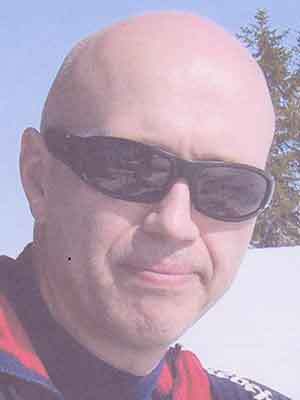
Arild was a professor at the Inland Norway University of Applied Sciences (INN University), Faculty of Social and Health Sciences, Elverum, Norway.

▪ Scientist, ranked chess player, debater and commentator. ▪ Research topics: Statistics, registries (birth & cancer), applied epidemiology, newborn, pregnancy health and outcomes, breastfeeding, cancer, sports medicine, ergonomics. ▪ Publications: 31 are listed in Pubmed for the period January 2004-July 2019.

Arild successfully defended his DrScient (PhD) research in 2005 at the Department of Community Medicine, UiT The Arctic University of Norway, Tromso, Norway. It involved multiple trips to the Kola Peninsula and the Arkhangelsk Region of Russia. In order to assess the potential impact of women working in the nickel refinery in the city of Monchegorsk, he pioneered the setting up of a retroactive birth registry. It eventually covered the period from 1973 to 2005 with 26 846 registered births [1]. Arild’s PhD publications employed this database to establish that: the annual perinatal mortality rate dropped from 20 to 10 per 1000 births in the period 1973–97; a relatively high prevalence of abortion and pelvic inflammatory disease (42.5% of the women worked in a nickel refinery); and little impact of maternal exposure to water-soluble nickel on malformations of the genital organs, musculoskeletal defects, and risk of small-for-gestational age. Arild’s Monchegorsk efforts led to the subsequent establishment of the Murmansk County Birth Registry (MCBR) that eventually covered all births (n = 52 806) in Murmansk County for the period 1 January 2006–31 December 2011 [2]. Multiple PhD students have used the MCBR database in their thesis publications. During his own graduate studies, Arild and colleagues from the Arkhangelsk Oncological Hospital initiated the publication of data compiled in the Arkhangelsk Cancer Registry database [3].

Arild was on an official WHO mission from 1 May 2014 to 31 March 2015 in Jerusalem as a Health Information Officer. In this context, he served as a designated expert for the Norwegian Refugee Council (NORCAP). Furthermore, his care and support for Russian orphans was admirable. Arild was also engaged in the circumpolar health community through the Nordic Society, a subgroup of the International Union of Circumpolar Health (IUCH).

Arild was multi-lingual, adding English and Russian to his Norwegian mother tongue. He was inquisitive, willing to work with others, stimulated those working with him and was a good travel companion. He respected the views of others while defending his own perspectives vigorously. Arild enjoyed life fully and will be sorely missed.
